# Gut bacterial ClpB-like gene function is associated with decreased body weight and a characteristic microbiota profile

**DOI:** 10.1186/s40168-020-00837-6

**Published:** 2020-04-30

**Authors:** María Arnoriaga-Rodríguez, Jordi Mayneris-Perxachs, Aurelijus Burokas, Vicente Pérez-Brocal, Andrés Moya, Manuel Portero-Otin, Wifredo Ricart, Rafael Maldonado, José-Manuel Fernández-Real

**Affiliations:** 1 Department of Diabetes, Endocrinology and Nutrition, Dr. Josep Trueta University Hospital, Carretera de França s/n, 17007 Girona, Spain; 2 Nutrition, Eumetabolism and Health Group, Girona Biomedical Research Institute (IdibGi), Girona, Spain; 3 CIBER Fisiopatología de la Obesidad y Nutrición (CIBEROBN), Madrid, Spain; 4 Department of Medical Sciences, Faculty of Medicine, University of Girona, Girona, Spain; 5 Laboratory of Neuropharmacology, Department of Experimental and Health Sciences, Universitat Pompeu Fabra, Barcelona, Spain; 6 Present address: Institute of Biochemistry, Life Sciences Center, Vilnius University, Saulėtekio av. 7, LT-10257 Vilnius, Lithuania; 7 Department of Genomics and Health, Foundation for the Promotion of Health and Biomedical Research of Valencia Region (FISABIO-Public Health), Valencia, Spain; 8 Biomedical Research Networking Center for Epidemiology and Public Health (CIBERESP), Madrid, Spain; 9 Institute for Integrative Systems Biology (I2SysBio), University of Valencia, Spanish National Research Council (CSIC-UVEG), Valencia, Spain; 10 Metabolic Pathophysiology Research Group, Lleida Biomedical Research Institute (IRBLleida), Universitat de Lleida, Lleida, Spain; 11 Hospital del Mar Medical Research Institute (IMIM), Barcelona, Spain

**Keywords:** Microbiome, Bacterial gene function, Body weight regulation, Obesity

## Abstract

**Background:**

The chaperone ClpB, a bacterial protein, is a conformational antigen-mimetic of α-melanocyte-stimulating hormone (α-MSH) implicated in body weight regulation in mice. We here investigated the potential associations of gut bacterial ClpB-like gene function with obesity status and gut microbiota in humans.

**Results:**

Gut microbiota ClpB KEGG function was negatively associated with body mass index, waist circumference, and total fat mass (DEXA). The relative abundance (RA) of several phyla and families directly associated with ClpB was decreased in subjects with obesity. Specifically, the RA of *Rikenellaceae*, *Clostridiaceae* and not assigned *Firmicutes* were lower in subjects with obesity and positively associated with gut bacterial ClpB-like gene function (not assigned *Firmicutes* (*r =* 0.405, FDR = 2.93 × 10−2), *Rikenellaceae* (*r =* 0.217, FDR = 0.031), and *Clostridiaceae* (*r =* 0.239, FDR = 0.017)). The gut bacterial ClpB-like gene function was also linked to specific plasma metabolites (hippuric acid and 3-indolepropionic acid) and fecal lupeol. The α-MSH-like epitope similar to that of *Escherichia coli* ClpB was also identified in some sequences of those bacterial families.

After fecal transplantation from humans to mice, the families that more contributed to ClpB-like gene function in humans were also associated with ClpB-like gene function in mice after adjusting for the donor’s body mass index (not assigned *Firmicutes* (*r =* 0.621, *p =* 0.003), *Prevotellaceae* (*r =* 0.725, *p =* 4.1 × 10^−7^), *Rikenellaceae* (*r =* 0.702, *p =* 3.9 × 10^−4^), and *Ruminococcaceae* (*r =* 0.526, *p =* 0.014)). *Clostridiaceae* (*r =* − 0.445, *p =* 0.038) and *Prevotellaceae* RA (*r = −* 0.479, *p =* 0.024) and were also negatively associated with weight gain in mice. The absolute abundance (AA) of *Prevotellaceae* in mice was also positively associated with the gut bacterial ClpB-like gene function in mice. DESeq2 identified species of *Prevotellaceae*, both negatively associated with mice’ weight gain and positively with gut bacterial ClpB-like gene function.

**Conclusions:**

In summary, gut bacterial ClpB-like gene function is associated with obesity status, a specific gut microbiota composition and a plasma metabolomics profile in humans that could be partially transplanted to mice.

Video Abstract

## Background

Obesity is rapidly becoming a global epidemic and its management is a priority challenge to halting this tendency. The role of the microbiota in the pathophysiology of obesity [1] and related complications [2] is increasingly recognized. The microbiota-gut-brain axis has been associated with the regulation of metabolism, energy balance, adiposity, central appetite, and food reward [3, 4]. Gut microbiota could modulate intestinal satiety and hunger hormones such as g*lucagon-like peptide-1* (GLP-1), peptide YY (PYY), and ghrelin [5] that impact on anorexigenic and orexigenic pathways, and also proopiomelanocortin (POMC) and neuropeptide Y (NPY)/agouti-related protein (AgRP) neurons, finally integrated in the hypothalamus [6].

Host energy homeostasis could be regulated by the bacterial production of metabolites and neurotransmitters or even by energy harvesting of their own bacterial metabolism [7, 8]. Recently, it has also been observed that bacterial proteins which directly act in the brain via vagal stimulation or indirectly through immune-neuroendocrine mechanisms have an important role in this process [7, 9]. One of these bacterial proteins, the caseinolytic peptidase B protein homolog (ClpB), has been identified as a conformational antigen-mimetic of α-melanocyte-stimulating hormone (α-MSH) [10]. The α-MSH is an amino-acid derived from POMC that activates the melanocortin-4 receptor (MC4R) expressed in the hypothalamic paraventricular nucleus promoting the anorexigenic pathway and therefore, regulating satiety, energy, blood pressure, and growth [11].

Tennoune et al. found that ClpB-immunized mice produced anti-ClpB IgG cross-reactive with α-MSH, influencing food intake and body weight. In addition, these authors reported increased plasma levels of an antibody anti-ClpB in patients with anorexia nervosa, bulimia, and binge-eating disorder [10]. On the other hand, Breton et al. described how regular nutrient provision stabilized exponential growth of *Escherichia coli*, with the stationary phase occurring 20 min after nutrient supply [9]. ClpB was upregulated in the *E. coli* stationary phase and plasma ClpB was proportional to ClpB DNA in feces, stimulating the firing rate of hypothalamic POMC neurons [9]. Furthermore, the administration of a probiotic, *Hafnia alvei*, a ClpB-producing bacterium, decreased fat mass and food intake in *ob/ob* and after a high-fat diet in mice [12].

To our knowledge, there is little evidence assessing ClpB gene function in subjects with obesity. Current information points to a lower gene richness in individuals with obesity and a negative association with body mass index of bacteria belonging to genera of *Enterobacter*, *Klebsiella*, and *Hafnia* in sillico analysis using the MetaHIT database [12]. Therefore, our main aim was to evaluate gut bacterial ClpB-like gene function in subjects with obesity compared to controls and assess the potential role of the microbiota composition and microbial-derived compounds in the modulation of body weight.

## Results

### Gut bacterial ClpB-like gene function is associated with decreased body weight in humans

**Fig. 1:**
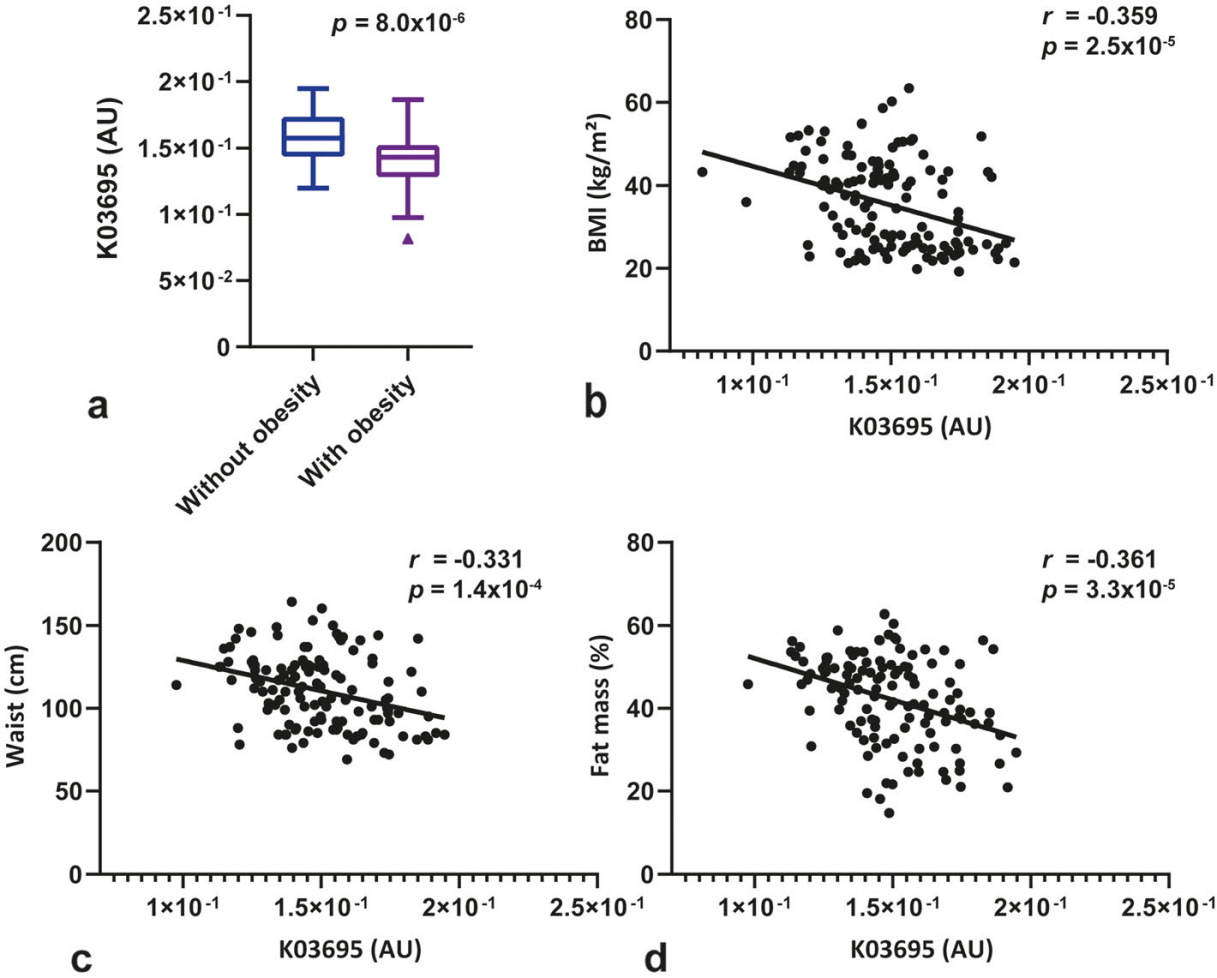
Gut bacterial ClpB-like gene function is associated with decreased body weight in humans. **a** Box plot showing the differences of gut bacterial ClpB-like gene function (K03695, KEGG annotation) between subjects without (body mass index, BMI 18.5–30 kg/m^2^) and with obesity (BMI ≥ 30 kg/m^2^). **b** Scatterplot displaying the relationship between BMI and gut bacterial ClpB-like gene function. **c** Scatterplot of the relationship between waist circumference and gut bacterial ClpB-like gene function. **d** Scatterplot of the relationship between the percentage of total fat mass assessed by DEXA and gut bacterial ClpB-like gene function

A consecutive series of 131 subjects, 76 of them with obesity and their respective paired by sex and age controls, was studied (Table 1). The detection of the gut bacterial ClpB-like gene function assessed using shotgun metagenomic analysis of fecal microbiota using the KEGG annotation, K03695 (subcategory (sc): aging/protein families: genetic information processing; pathway (p): longevity regulating pathway—multiple species [PATH:ko04213]/chaperones and folding catalysts [BR:ko03110]; annotation description (ad): ClpB ATP-dependent Clp protease ATP-binding subunit ClpB; and annotation (a): K03695). This function was significantly lower in subjects with obesity (Fig. 1a; Table 1). In addition, this ClpB-like gene function was negatively associated with body mass index (Fig. 1b), waist circumference (Fig. 1c), and total fat mass (Fig. 1d). Other KEGG functions were also negatively associated with body mass index but not so strongly, such as K01358 (*r = -*0.277, *p =* 0.001; sc: cell growth and death/aging/protein families: metabolism; p: cell cycle—Caulobacter [PATH:ko04112]/longevity regulating pathway—worm [PATH:ko04212]/peptidases [BR:ko01002]; ad: ClpP, CLPP ATP-dependent Clp protease, protease subunit [EC:3.4.21.92]; a: K01358) and K01419 (*r = −* 0.276, *p =* 0.001, sc: protein families: metabolism; p: peptidases [BR:ko01002]; ad: hslV, clpQ ATP-dependent HslUV protease, peptidase subunit HslV [EC:3.4.25.2]; and a: K01419).

**Table 1:** Clinical characteristics of the human samples

	Without obesity	With obesity	
	(*n* = 55)	(*n* = 76)	*p v*alue
Females *n* (%)	37 (67.3)	52 (68.4)	0.519
Age (years)	53.7 [17.4]	48.6 [15.7]	0.188
BMI (kg/m^2^)	24.9 (2.5)	43.2 (6.8)	< 0.001
Waist (cm)	89.7 (9.6)	126.2 (14.1)	< 0.001
Fat total (%)	32.5 (7.5)	49.6 (5.6)	< 0.001
Android fat (%)	32.6 (10.5)	56.5 (5.4)	< 0.001
Gynoid fat mass (%)	37.5 [15.6]	50.6 [8.7]	< 0.001
SBP (mmHg)	124.0 (16.1)	139.2 (19.3)	< 0.001
DBP (mmHg)	71.1 (11.0)	78.0 (10.9)	0.001
Glucose (mg/dL)	95.0 [13.0]	96.0 [13.5]	0.284
HbA1c (%)	5.5 (0.3)	5.6 (0.3)	0.016
Cholesterol (mg/dL)	200.0 [48.0]	183.5 [57.0]	0.043
LDL-cholesterol (mg/dL)	119.0 [48.0]	112.0 [58.8]	0.634
HDL-cholesterol (mg/dL)	63.9 (17.5)	53.5 (12.0)	0.005
Triglycerides (mg/dL)	79.0 [39.0]	111.5 [75.0]	< 0.001
hsCRP (mg/dL)	0.7 [0.9]	4.9 [7.2]	< 0.001
K03695 (AU)	1.6 × 10^−1^ (3.0 × 10^−2^)	1.4 × 10^−1^ (2.4 × 10^−2^)	8.0 × 10^−6^

Results are expressed as number and frequencies for categorical variables, mean and standard deviation (SD) for normal distributed continuous variables, and median and interquartile range [IQR] for non-normal distributed continuous variables. To determine differences between study groups, we used χ^2^ for categorical variables, unpaired Student’s *t* test in normal quantitative, and Mann-Whitney *U* test for non-normal quantitative variables. *BMI* body mass index, *SBP* systolic blood pressure, *DBP* diastolic blood pressure, *HbA1c* glycated hemoglobin, *LDL* low density lipoprotein, *HDL* high density lipoprotein, *hsCRP* high-sensitive C-reactive protein, *AU* arbitrary units

As diet is a well-known modifier of bacterial ecosystems, we explored which dietary components, by focusing on macronutrients and fiber, were linked to gut bacterial ClpB-like gene function (K03695). As a crude measure, total energy intake was negatively associated with ClpB-like gene function (*r = −* 0.189, *p =* 0.041). After adjusting for total energy intake and body weight, gut bacterial ClpB-like function was directly associated with proteins (*r =* 0.200, *p =* 0.032), carbohydrates (*r =* 0.224, *p =* 0.015), lipids (*r =* 0.303, *p =* 0.001), and fiber intake (*r =* 0.193, *p =* 0.037). Nevertheless, in multiple linear regression models, with gut bacterial ClpB-like gene function (K03695) as dependent variable and body mass index, sex, age, total energy intake, and each macronutrient or fiber, as independent variables, only body mass index (e.g., model with proteins, *β* = *−* 0.345, *p* = 4.3 × 10^−4^) remained significantly associated with gut bacterial ClpB-like gene function.

### Gut bacterial ClpB-like gene function and microbiota composition and metabolites

**Fig. 2:**
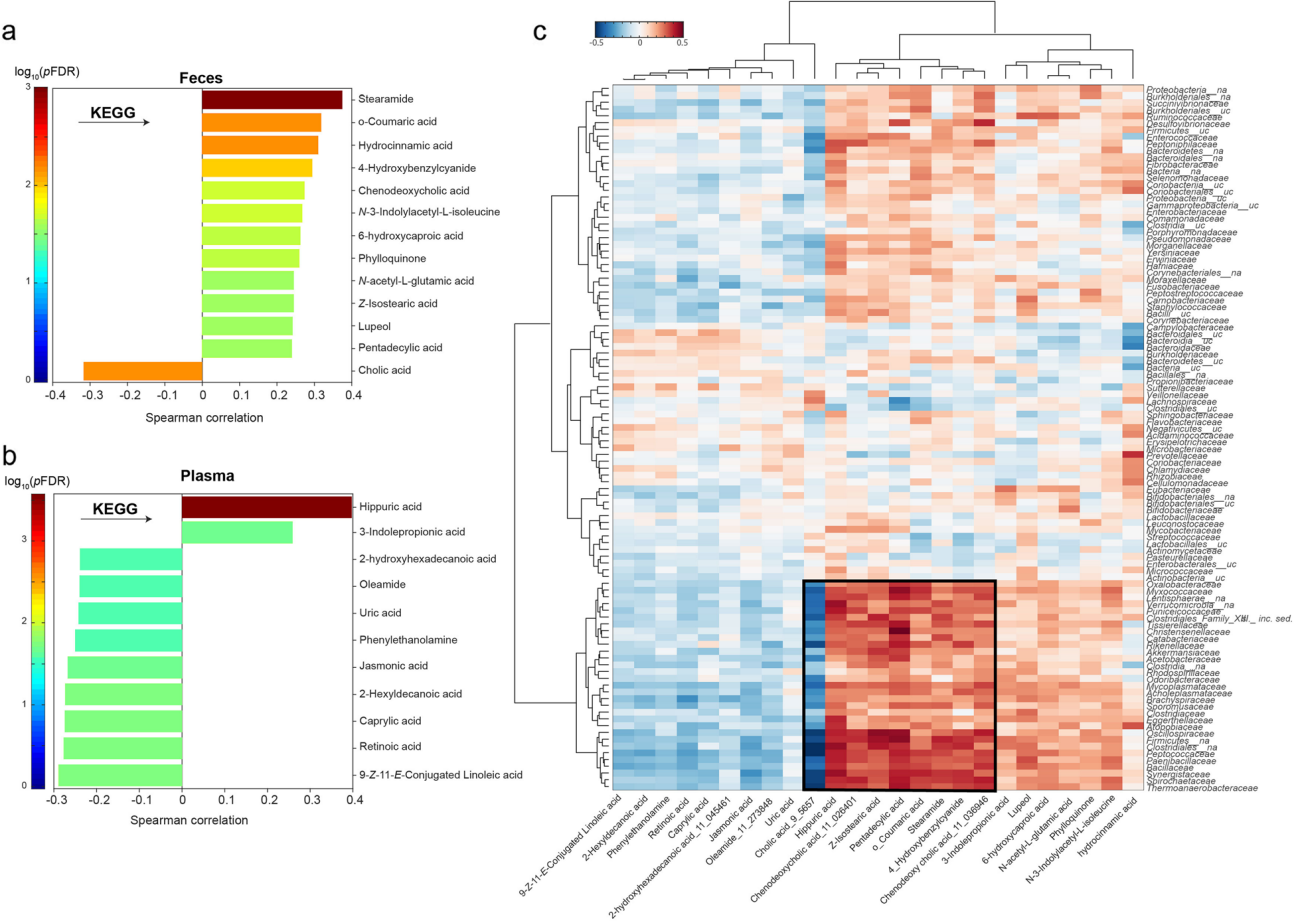
Gut bacterial ClpB-like gene function and microbiota metabolites. **a** Significant O-PLS correlations coefficients for the model between gut bacterial ClpB-like gene function (K03695, KEGG annotation) and metabolites in feces (data in feces was previously normalized using the probabilistic quotient normalization (PQN)). **b** Significant O-PLS correlation coefficients for the model between gut bacterial ClpB-like gene function (K03695, KEGG annotation) and metabolites in plasma. **c** Heatmap showing the Spearman correlation coefficients and associated *p* values adjusting by FDR between significant metabolites in plasma and feces and bacterial families. Hierarchical clustering analysis was performed using Ward linkage and Euclidean distance. A cluster with the strongest correlations is highlighted with a black square

Gut bacterial ClpB-like gene function was found to be associated with bacterial metabolites in feces and plasma samples. To note, ClpB was positively associated with hippuric acid (*r =* 0.398, *p =* 2.85 × 10^−4^) and 3-indolepropionic acid (*r =* 0.259, *p =* 0.034) in plasma and lupeol in feces (*r =* 0.242, *p =* 0.039) and negatively with cholic acid in feces (*r = −* 0.317, *p =* 0.008) (Fig. 2a, b) and a common gut microbiota ecosystem was simultaneously associated with these metabolites and ClpB-like gene function (Fig. 2c).

**Fig. 3:**
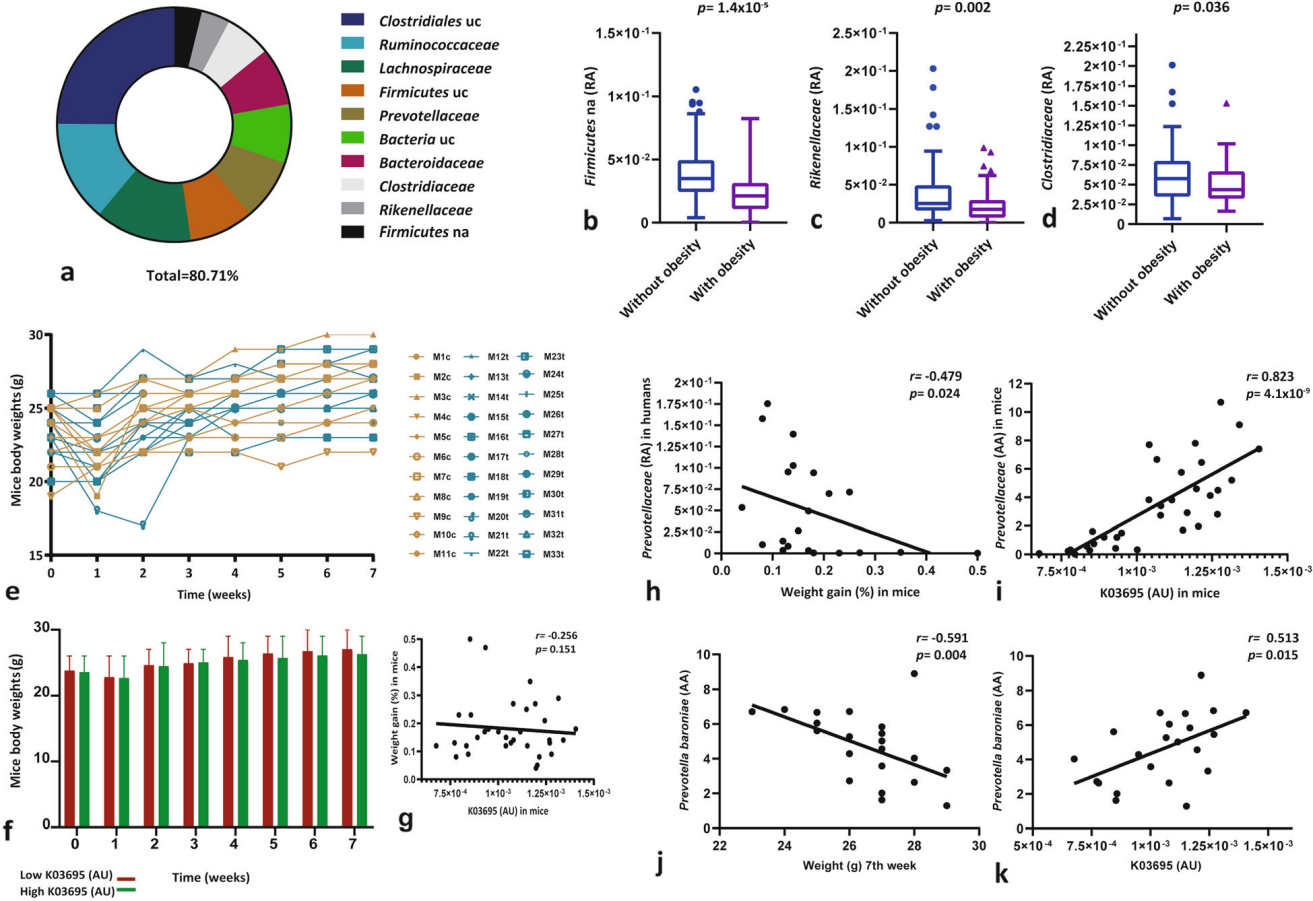
Gut bacterial ClpB-like gene function and metagenomics. **a** Top bacterial taxa at the family level contributing reads to the gut bacterial ClpB-like gene function, in total relative figures per sample (uncultured, uc; not assigned, na). **b**–**d** Box plots showing the differences of some of the top ten bacterial families (relative abundance, RA) contributing to the gut bacterial ClpB-like gene function in subjects with and without obesity. **e** Mice body weights after gut microbiota transplantation. Controls (mouse, M; control, c) are shown in yellow (*n* = 11) and mice which received microbiota from human donors (transplant, t) are shown in blue (*n* = 22). **f** Bar chart showing mean and standard deviation mice body weights, regarding the median of the gut bacterial ClpB-like gene function detected in mice, K03695 (arbitrary units, AU). **g** Scatterplot showing the relationship between percent weight gain in mice (calculated as (body weight final (g)—body weight initial (g))/body weight initial (g)*100) and gut bacterial ClpB-like gene function, K03695 (AU), in mice. **h** Scatterplot showing the relationship between percent weight gain in mice and *Prevotellaceae* RA in humans. **i** Scatterplot showing the relationship between gut bacterial ClpB-like gene function (K03695, KEGG annotation) and *Prevotellaceae* absolute abundance (AA) in mice. **j** Scatterplot showing the relationship between the final weight (7th week) in mice and *Prevotellaceae baroniae* AA in humans. **k** Scatterplot showing the relationship between gut bacterial ClpB-like gene function (K03695, KEGG annotation) in mice and *Prevotellaceae baroniae* AA in human donors

The bacterial families with the highest number of reads that contributed most to the ClpB-like gene function were the following: *Clostridiaceae*, *Ruminococaceae*, *Lachnospiraceae*, uncultured and not assigned *Firmicutes*, and uncultured *Clostridiales* within the *Firmicutes* phylum and *Prevotellaceae*, *Bacteroidaceae*, and *Rikenellaceae* within *Bacteroidetes* phylum (Fig. 3a; Additional file 1). Interestingly, while the relative abundance (RA) of not assigned *Firmicutes* (Fig. 3b), *Rikenellaceae* (Fig. 3c), and *Clostridiaceae* (Fig. 3d) families were lower in subjects with obesity, these families were also positively associated with gut bacterial ClpB-like gene function (not assigned *Firmicutes* (*r* = 0.405, FDR = 2.93 × 10^−2^), *Rikenellaceae* (*r* = 0.217, FDR = 0.031), and *Clostridiaceae* (*r* = 0.239, FDR = 0.017)). Importantly, the associations between ClpB-like gene function and some families *Clostridiaceae* (*r =* 0.352, *p =* 4.2 × 10^−5^), *Ruminococaceae* (*r =* 0.284, *p =* 0.001), not assigned *Firmicutes* (*r =* 0.523, *p =* 2.1 × 10^−10^), and uncultured *Firmicutes* (*r =* 0.367, *p* = 1.8 × 10^−5^) remained significant even after adjusting for body mass index.

Focusing on the α-MSH molecular mimicry, the families that most contributed to the ClpB-like gene function (Fig. 3a) and were also detected in relative lower abundance in subjects with obesity (Fig. 3b–d), such as not assigned *Firmicutes*, *Rikenellaceae*, and *Clostridiaceae* showed 48,192 and 175 sequences with 100% homology with α-MSH epitope of *E. coli* with a total mean of identity of 82.13%, 92.25%, and 81.36%, respectively (Additional files 2-3). Other families, that also have an important contribution to ClpB-like gene function, such as uncultured *Clostridiales*, *Ruminococcaceae*, *Lachnospiraceae*, uncultured *Firmicutes*, *Prevotellaceae*, uncultured *Bacteria*, and *Bacteroidaceae* (Fig. 3a) presented 1068, 766, 855, 265, 262, 149, and 431 sequences with a total homology with motif in *E. coli* and a total mean of identity of 89.04%, 85.04%, 88.28%, 86.44%, 95.78%, 90.01%, and 93.64%, respectively (Additional files 2-3).

Furthermore, the bacterial families whose reads more contributed to the ClpB-like gene function were also associated with some bacterial metabolites (Fig. 2c). Remarkably, a cluster of metabolites and not assigned *Firmicutes*, *Rikenellaceae*, and *Clostridiaceae*, among others, were identified (Fig. 2c).

### Gut microbiota transplantation in mice

Finally, we tested the gut bacterial ClpB-like gene function in 33 mice with no significant differences in initial body weight (Fig. 3e). After antibiotic treatment, 11 mice were orally gavaged with vehicle and 22 with fecal material from 22 subjects paired in age and sex. ClpB-like gene function in mice showed a trend to be negatively associated with body weight gain as the time of the experiment passed (Fig. 3f) and also a negative tendency to be associated with percent weight gain at the end of the experiment (Fig. 3g). ClpB-like gene function in humans was not significantly associated with ClpB-like gene function in mice. Nevertheless, the families that more contributed to ClpB-like gene function in humans were also associated with ClpB-like gene function in mice after adjusting for the donor’s body mass index, such as not assigned *Firmicutes* (*r =* 0.621, *p =* 0.003), uncultured *Firmicutes* (*r =* 0.529, *p =* 0.014), *Prevotellaceae* (*r =* 0.725, *p =* 4.1 × 10^−7^), *Rikenellaceae* (*r =* 0.702, *p =* 3.9 × 10^−4^), and *Ruminococcaceae* (*r =* 0.526, *p =* 0.014). Furthermore, two of the bacterial families that more contributed to the gut bacterial ClpB-like function in humans, *Clostridiaceae* (*r =* − 0.445, *p =* 0.038) *and Prevotellaceae* (*r =* − 0.479, *p =* 0.024) were negatively associated with weight gain in mice (Fig. 3h). The absolute abundance (AA) of *Prevotellaceae* in mice was also positively associated with the gut bacterial ClpB-like gene function in mice (Fig. 3i). A deeper analysis using DESeq2 [13] identified species of *Prevotellaceae*, both negatively associated with mice’ weight in the 7th week of follow-up (Fig. 3j) and positively with gut bacterial ClpB-like gene function (Fig. 3k).

## Discussion

Gut bacterial ClpB-like gene function in feces (KEGG annotation: K03695) was associated with decreased body weight, central obesity, and total fat mass in humans. Accordingly, this gut bacterial function was found in lower relative abundance in subjects with obesity. These results are in line with previous evidence in mouse models and in sillico findings. In a recent study, the administration of a probiotic, *Hafnia alvei*, a ClpB-producing bacterium reduced fat mass and food intake in *ob/ob* and after a high-fat diet in mice [12] and also improved their metabolic profile [14]. In this study, the enterobacterial ClpB gene was also tested in sillico analysis of fecal metagenomes from the MetaHIT database [12]. To our knowledge, our study sets up the first evidence of the association of ClpB gene function and obesity in a clinical setting.

The caseinolytic protease B (ClpB) protein described in *E. coli* is an ATP-dependent ring forming chaperone that mediates the resolubilization of aggregated proteins [15]. This gut bacteria ClpB protein has been identified as a conformational antigen-mimetic of α-MSH that could activate the MC4R in the hypothalamus ultimately promoting the anorexigenic pathway [10]. In that sense, autoantibodies that interreacted with the α-MSH were found elevated in subjects with anorexia and bulimia nervosa [16, 17], and plasma levels of ClpB were proportional to ClpB DNA in feces [9]. In our study, metagenomic analysis of fecal microbiota revealed that the RA of *Rikenellaceae*, *Clostridiaceae* and not assigned *Firmicutes* were positively associated with ClpB gene function and were detected in lower relative abundance in subjects with obesity. Tennoune et al. firstly demonstrated the α-MSH mimetic ClpB protein in *E. coli* from the phylum *Proteobacteria* [10]. Other sources of α-MSH-like epitope of *E.coli* ClpB identified in sillico analysis were bacteria belonging to the genera *Escherichia*, *Salmonella*, *Shigella*, *Klebsiella*, *Enterobacter*, *Citrobacter*, *Cronobacter*, *and Hafnia*, and in that search, the ClpB appeared to be specific to the family *Enterobacteriaceae* and in some *Brassicaceae* [18]. However, except for *E. coli* ClpB, we did not find studies that have tested the properties of this protein in the previous genera described. Other bacteria that present sequence homology in at least five amino acids with α-MSH have been reported, such as bacteria belonging to the phyla *Bacteroidetes*, *Actinobacteria* (*Bifidobacterium longum*, *Frankia*), *Firmicutes* (*Bacillus cereus*, *Clostridium tetani*), or *Proteobacteria* (*Helicobacter*) [19]. In our study, we have identified that the sequences of the bacterial families which more contributed to gut bacterial ClpB-like gene function presented the motif of α-MSH-like epitope of *E.coli* ClpB, suggesting that this mechanism could be shared with other bacterial families and might be explain the role on body weight regulation. However, the presence of amino-acid sequence homology does not imply the same molecular function and experimental studies are needed to test the α-MSH mimicry of the different ClpB proteins from other microbial organisms [18]. Nonetheless, there is some evidence that gut bacterial ClpB-like gene function, either by the same α-MSH-like epitope of ClpB *E.coli* with an effect on the anorexigenic pathway [18] or by others ClpB fragments with other unknown routes implicated, seems to have a role in the pathophysiology of obesity attributed to the gut microbiota.

In addition, we found a relationship between the diet and the detection of ClpB-like gene function in humans. In general, a negative association was found between total energy intake and gut bacterial ClpB-like gene function. This data is concordant with previous evidence in mice in which the dietary restriction induced an increase of ClpB [20]. On the contrary, we observed positive associations between ClpB-like gene function and different macronutrients and fiber. Proteins have been identified as the main contributors to the ClpB *E.coli* levels in in vitro studies [21]. In our study, positive relationships were found not only with total protein intake but also with fiber intake and the rest of macronutrients. However, the strength of the body mass index appeared to be more important than the role of each macronutrient separately. Interestingly, ClpB-like gene function was associated with some metabolites in plasma such as hippuric acid and 3-indolepropionic acid, or lupeol and cholic acid in feces. The direction of the relationships is in line with current evidence. Higher levels of hippuric acid have been associated with a beneficial metabolic profile, decreased fasting plasma glucose and insulin secretion [22]. Similar observations have been made regarding 3-indolepropionic acid [23]. Lupeol has exhibited antioxidant, anti-inflammatory, anti-hyperglycemic, hypolipemiant, and anti-mutagenic properties [24].

In the animal experiment, microbial families that seem to contribute to the gut bacterial ClpB-like gene function, such as *Prevotellaceae* and *Clostridiaceae* were negatively associated with weight gain during the 7 weeks of follow-up. Despite not directly observing a negative significant association between gut bacterial ClpB-like gene function and weight gain in mice, it can be seen a tendency as the time of the experiment passed. Maybe, if the duration of the study would have been longer or the mice sample size larger, a significant association would be reached. In any case, the direction of the association between gut bacterial ClpB-like gene function and body weight seems to be shared between humans and mice. This would be the first study investigating the effects of microbiota transplantation from humans to mice evaluating ClpB function. Previous reports in mice were based on chronic intragastric delivery of *E.coli* [10] and *H. alvei* [12].

## Conclusions

ClpB protein has been previously studied in animal models [9, 10]. Antibody anti-ClpB was investigated in a single human study [10]. This is the first study, to our knowledge, evaluating gut bacterial ClpB-like gene function in human subjects linked to obesity status, gut bacterial ecosystem, and diet. The findings of the current study show that a bacterial ecosystem enriched in ClpB-like gene function that was negatively associated with body mass index, waist circumference, and fat mass, and detected in lower abundance in subjects with obesity. We speculate that this gut bacterial ClpB-like function could lead to increased satiety and decreased fat mass in the long term.

## Methods

### Clinical study

#### Recruitment of study subjects

From January 2016 to October 2017, a cross-sectional case-control study was undertaken in the Endocrinology Department of Josep Trueta University Hospital. We included consecutive subjects with obesity (body mass index, BMI ≥ 30 kg/m^2^) and age- and sex-matched nonobese subjects (BMI 18.5–30 kg/m^2^), with an age range of 27.2–66.6 years. Exclusion criteria were: type 2 diabetes mellitus, chronic inflammatory systemic diseases, acute, or chronic infections in the previous month; use of antibiotic, antifungal, antiviral, or treatment with proton-pump inhibitors; severe disorders of eating behavior or major psychiatric antecedents; or excessive alcohol intake (≥ 40gOH/day in women or 80gOH/day in men). The institutional review board—Ethics Committee and the Committee for Clinical Research (CEIC) of Dr. Josep Trueta University Hospital (Girona, Spain)—approved the study protocol and informed written consent was obtained from all participants.

#### Clinical and laboratory parameters

Body composition was assessed using a dual energy x-ray absorptiometry (DEXA, GE lunar, Madison, Wisconsin).

#### Dietary pattern

The dietary characteristics of the subjects were collected in a personal interview using a validated food-frequency questionnaire [25].

### Extraction of fecal genomic DNA and whole-genome shotgun sequencing

Total DNA was extracted from frozen human stools using the QIAamp DNA mini stool kit (Qiagen, Courtaboeuf, France). Quantification of DNA was performed with a Qubit 3.0 fluorometer (Thermo Fisher Scientific, Carlsbad, CA, USA), and 1 ng of each sample (0.2 ng/μL) was used for shot gun library preparation for high-throughput sequencing, using the Nextera DNA Flex Library Prep kit (Illumina, Inc., San Diego, CA, USA) according to the manufacturers’ protocol. Sequencing was carried out on a NextSeq 500 sequencing system (Illumina) with 2 × 150-bp paired-end chemistry, at the facilities of the Sequencing and Bioinformatic Service of the FISABIO (Valencia, Spain).

The obtained input fastq files were decompressed, filtered, and 3 ends-trimmed by quality, using prinseq-lite-0.20.4 program [26] and overlapping pairs were joined using FLASH-1.2.11 [27]. Fastq files were then converted into fast files, and human and mouse host reads were removed by mapping the reads against the GRCh38.p11, reference human genome (Dec 2013), and GRCm38.p6, reference mouse genome (Sept 2017), respectively, by using bowtie2-2.3.4.3 [28] with end-to-end and very sensitive options.

Next, functional analyses were carried out by assembling the non-host reads into contigs by MEGAHIT v1.1.2 [29] and mapping those reads against the contigs with bowtie 2. Reads that did not assemble were appended to the contigs. Next, the program Prodigal v2.6.342 [30] was used for predicting codifying regions. Functional annotation was carried out with HMMER [31] against the Kyoto Encyclopedia of Genes and Genomes (KEGG) database, version 2016 [32] to obtain the functional subcategory, route, and annotation of the genes. An in-house pipeline, consisting of a series of consecutive customized scripts, implemented by the statistical package R 3.1.0 [33], coordinated and concatenated by shell commands, was applied to the functionally annotated reads to first parse the HMMER table, and next to get the best annotation per open reading frame (ORF) and to identify which ORFs were annotated, followed by getting the nucleotides and amino acids sequences. Next, it allowed us to get non-singleton contigs with annotated ORFs (annotated contigs), add the annotation for each ORF, get the reads inside that annotated ORFs, count the alignments per annotation, count alignment per category (a specific annotation can belong to multiple categories), and finally build a contingency table to which descriptions were added.

Taxonomic annotation was implemented with Kaiju v1.6.2 [34] on the human and mouse-free reads. Addition of lineage information was added, counting of taxa and generation of an abundance matrix for all samples were performed using the package R.

#### Molecular mimicry

The mimetic amino acid motif of the α-MSH peptide (RWGKPV) was first aligned to the ClpB from *E. coli* str K12 (accession number NP_417083.1). Then, the predicted ORFs from each sequence were aligned with Clustal Omega [35] to the previous two sequences. Those that encompassed the region that contained the epitope-like sequence were filtered, discarding reads which not included it. Sequences beyond 30 amino acids before and after the aligned α-MSH peptide were trimmed and 40 alignments were plotted. R scripts [33] allowed us to create a table with the aligned motif and calculate the identity between the *E. coli* and the microbial counterparts in the sequences, as well as, assign the taxonomy, at family level, to each contig or single read. The average identity of the epitope in each bacterial family compared to the motif in *E. coli* as well as the distribution of families through the sequences according to their degree of homology were also calculated with R package [33].

### Metabolomics analyses

For non-targeted metabolomics analysis, metabolites were extracted from fecal samples with methanol according to previously described methods [36], by using phenylalanine-C13 as internal standard. After 1 h at − 20 °C, methanol supernatants were centrifuged at 12000*g* for 3 min and evaporated using a Speed Vac (Thermo Fisher Scientific, Barcelona, Spain). The resulting pellets were resuspended in water with 0.4% acetic acid/methanol (50/50).

We used an ultra-high-pressure liquid chromatography (UHPLC) with an Agilent 1290 LC system coupled to an electrospray-ionization quadruple time of flight mass spectrometer (Q-TOF) 6520 instrument (Agilent Technologies, Barcelona, Spain). A column with 1.8 μm particle size was employed and we performed the preliminary identification of differential metabolites by using the database PCDL from Agilent (Agilent Technologies, Barcelona, Spain), which uses retention times, exact mass, and isotope distribution in a standardized chromatographic system as an orthogonal searchable parameter to complement accurate mass data (AMRT approach) according to previously published works [37].

### Animal procedures

Male C57BL/6 J mice (Charles River, France), weighing 29–26 g at the beginning of the experiment were used in this study. Mice were housed individually in controlled laboratory conditions with the temperature maintained at 21 ± 1 °C and humidity at 55 ± 10% during all the study. Animal procedures were conducted in strict accordance with the guidelines of the European Communities Directive 86/609/EEC regulating animal research and were approved by the local ethical committee (CEEA-PRBB). All the experiments were performed under blinded conditions.

Then, mice were given a cocktail of ampicillin and metronidazole, vancomycin (all at 500 mg/L), ciprofloxacin HCl (200 mg/L), and imipenem (250 mg/L) once daily for 14 consecutive days in drinking water, as previously described [38]. Seventy-two hours later, animals were colonized via daily oral gavage of donor microbiota (150 μL) for 3 days. Animals were orally gavaged with fecal material from healthy volunteers (*n* = 22). To offset potential confounder and/or cage effects and to reinforce the donor microbiota phenotype, booster inoculations were given twice per week throughout the study. Animals were maintained on normal mouse chow diet (Rat and Mouse No. 1 Maintenance Diet, Special Diet Services, Essex, UK) and water ad libitum, and were weighted every week during 7 weeks. Food intake was the same in the two groups. At the end of the study, the animals were consecutively sacrificed. The cecum was removed, weighted, and stored, and the feces collected and stored at − 80 °C for microbiota analysis.

### Statistical analysis

Firstly, normal distribution and homogeneity of variances were tested. Results are expressed as number and frequencies for categorical variables, mean and standard deviation (SD) for normal distributed continuous variables, and median and interquartile range [IQR] for non-normal distributed continuous variables. To determine differences between study groups, we used χ^2^ for categorical variables, unpaired Student’s *t* test in normal quantitative, and Mann-Whitney *U* test for non-normal quantitative variables. Nonparametric Spearman analysis was used to determine the correlation between quantitative variables. These analyses were performed using SPSS version 24 (IBM Corp., Armonk, NY) and GraphPad Prism version 8.00 (GraphPad Software, La Jolla, CA, USA).

Metabolomics data were analyzed using in-house MATLAB scripts. Orthogonal projection to latent structures (O-PLS) using unit variance scaling was then applied to identify metabolites associated with the gut bacterial ClpB-like gene function. Metabolites were used as the descriptor matrices (X) and gut bacterial ClpB-like gene function was used as the response (Y). The predictive performance (Q^2^Y) of each model was calculated using a seven-fold cross-validation approach and model validity was established by permutation testing (1000 permutations). Significance of O-PLS correlation coefficients was adjusted by the Benjamini-Hochberg method (FDR) [39].

Bacterial taxa associated with the gut bacterial ClpB-like gene function in humans were identified using the DESeq2 R packages [13]. Taxa were previously filtered so that only those with more than 10 reads in at least two samples were selected.

## Supplementary information

**Additional file 1.** Top 10 bacterial taxa (family level) contributing reads to the gut bacterial ClpB-like function, in absolute and relative figures per sample.

**Additional file 2. **Alignments of the mimetic amino acid motif of the α-MSH, the ClpB from *E. coli* str K12 and the predicted ORFs from each sequence of the samples 1, 30 and 143.

**Additional file 3. **Alignments. Alignment motif of the α-MSH and percentage of identity between the *E. coli* motif and the identified microbial counterparts in each sequence. Bacterial family identity: Average identity of the epitope in each bacterial family compared to the motif in *E. coli* as well as the distribution of families through the samples according to their degree of homology.

## Data Availability

The data that support the findings of this study are available from the corresponding author upon reasonable request.

## Abbreviations

α-MSH: α-melanocyte-stimulating hormone
AA: Absolute abundance
AgRP: Agouti-related protein
BMI: Body mass index
ClpB: Caseinolytic peptidase B protein homolog
GLP-1: Glucagon-like peptide 1
MC4R: Melanocortin-4 receptor
NPY: Neuropeptide Y
POMC: Proopiomelanocortin
PYY: Peptide YY
RA: Relative abundance
