# Supplementary material for: Gut bacterial ClpB-like gene function is associated with decreased body weight and a characteristic microbiota profile
Source: Microbiome. 2020 Apr 30;8:59. doi: 10.1186/s40168-020-00837-6 (PMC7193372; doi:10.1186/s40168-020-00837-6)
Supplement: Supplementary file 3 — Additional file 2. Alignments of the mimetic amino acid motif of the α-MSH, the ClpB from E. coli str K12 and the predicted ORFs from each sequence of the samples 1, 30 and 143. [file 40168_2020_837_MOESM2_ESM.pdf]

```
alphaMSH.IRM-001              ..............................RW.GKPV.............................   6
Ecoli-ClpB.IRM-001            ATQ....LEGKTMRLLRNKVTDAEIAEVLARWTGIPVSRMMESEREKLLRMEQELHHRVIGQNEAVD 578
contig 11902.IRM-001 1        TQQKLHEMQGD.KAMIKEEVDAEDIADVVSRWTGIPVSKMLQSEKDKLLQLEAELHKRVIGQ..... 407
contig 20018.IRM-001 1        .......................EIANVISMMSGIPVQRMAQAEGIKLAGMKEDLQSKVIAQDTAIE  44
contig 25392.IRM-001 1        TQKKLRDMQGD.KAMIKEEVDAEDIADVVSRWTGIPVSKMLQSEKDKLLHLEEELHQRVIGQDEAIE 102
contig 29383.IRM-001 1        ............SQALGEKDVRDEIAEVVSRATGIPVSKMMQGERQKLLHMDEALSKRVVGQDQAVR  86
contig 32308.IRM-001 6        TQKQLRDMQGD.KAMIKEEVDAEDIADVVSRWTGIPVSKMLQSEKDKLLHLEEELHQRVIGQDEAIE 576
contig 35309.IRM-001 33       IQEDLKHKQGD.SAMIKEEVTAEDIADVVSRWTGIPVNKMLQSERDKLLHLEQELHLRVVGQDEAIA 576
contig 48006.IRM-001 1        AEAKAK.KDGSDDSLVHENVTEEEIAKIISRWTGIPVAKLSESE.......................  49
contig 55266.IRM-001 1        .......VKKEDLSLVHESVTEDEIARIVSKWTGIPVAKLTESERSKTLHLDQVLHKRVVGQDEGVE  60
contig 68680.IRM-001 1        ANESAEDKD...NKLLRTKVTEEEIAMIVGRWTGIPVSKLMESE.......................  50
contig 73341.IRM-001 1        ......................EDEIAKIISRWTGIPVAKLTESERNKTLHLDKILHERVVGQDEAVE  46
seq 66415.IRM-001 1           VKKKLADMKHG.ESLIREEVTSDDIAAVVSKWTGIPVNRMMQSERMKLLHLEEELHKR.........  77
seq 135437.IRM-001 1          EERNTKE...SDNRLVHEAVTDDEIAKIISRWTGIPVTRLTEGERTKLLSLEDELHKRVIGQDE...  86
seq 160587.IRM-001 1          .........TQDLSLVHESVTEDEIARIISRWTGIPVSKLNESERSKILH.................  41
seq 168518.IRM-001 1          ............PKLLRTSVGAEEIAEVVSRATGIPVSKMMQGERQKLLHMDEALSKRVVGQDQAVR  79
seq 284160.IRM-001 1          ..................EVDSDDIADVVSRWTGIPVSKMMQSEKDKLLRLESELHTRVIGQEEAIN  49
seq 327988.IRM-001 1          ANESAEDKD...NKLLRTKVTEEEIAMIVGRWTGIPVSKLMESERKKLLDLDKILHK..........  57
seq 357381.IRM-001 1          ..............................WTGIPVSRMLASEREKLLHMEEELHRRVVGQDLAIA  36
seq 446578.IRM-001 1          NQKKQ.......NE..IPELTTEDAAEVVSLWTQIPVTHLTKGDMERLRHLEKE.............  52
seq 607059.IRM-001 1          EERNTKE...SDNRLVHEAVTDDEIAKIISRWTGIPVTRLTEG........................  49
seq 671420.IRM-001 1          ......................DEEIARIVARWTGIPVSKLMEGEREKLLHLEDILHQRVIGQDEAVT  46
seq 724706.IRM-001 1          ......................ADEIADIVSKWTGIPVSKLAETEKEKILNLENTLKDRVKGQDEAVR  46
seq 1511914.IRM-001 1         ............PKLLRTSVGAEEIAEVVSRATGIPVSKMMQGERQKL..................  50
seq 1542764.IRM-001 1         ..................SVTDEEIARIVSRWTGIPVAKLNESERSKTLHLADELH...........  38
seq 1849904.IRM-001 1         ...............VHENVTEEEIAKIISRWTGIPVAKLSESERQKTLNLDETLHKRVVGQDE...  49
```

logo

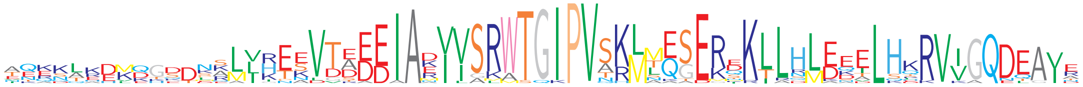

- X Asp, Glu
- X Arg, Lys, His
- X Phe, Tyr, Trp
- X Ala, Gly
- X Cys, Met
- X Ser, Thr
- X Asn, Gln
- X Leu, Val, Ile
- X Pro

```
alphaMSH.IRM-030          ..............................RW.GKPV..............................    6
Ecoli-ClpB.IRM-030        ATQLE....GKTMRLLRNKVTDAEIAEVLARWTGIPVSRMMESEREKLLRMEQELHHRVIGQNEAVD  578
contig 027087.IRM-030 1   ......S.TQGGAAMVREEVTADDIAEVVSRWTGIPVNRMMQSEREKLLHLEEELHKRVIGQDEAIT   60
contig 042720.IRM-030 1   KQAAAS..........EIKVNMDDVAKVIELWTGIPAVKIQETEFVKLAGLEAELKKKIIGQDE...   66
contig 051406.IRM-030 1   ........................IADVVSRWTGIPVSKMLQSEKDKLLHLEEELHQRVIGQDEAIE   43
contig 052055.IRM-030 1   ATQLE....GKTMRLLRNKVTDAEIAEVLARWTGIPVSRMMESEREKLLRMEQELHHRVIGQNEAVD  333
contig 056378.IRM-030 1   EEKLANE..KKEDSLLRDRVTDEEIARIVARWTGIPVEKLVEGEREKLLHLDDVLHKRVIGQDEAVT  271
contig 073530.IRM-030 1   IQEDLKH.KQGDSAMIKEEVTAEDIADVVSRWTGIPVNKMLQSERDKLLHLEQELHLRVVGQDEAIA  138
contig 078180.IRM-030 5   IQEDLKK.KQGDSALIKEEVTAEDIADVVSRWTGIPVSKMLQSEREKLLHLEDELHKRVIGQDEAIE  282
contig 089518.IRM-030 1   ..................EVDAEDIADVVSRWTGIPVSKMLQSEKEKLLHLEDELHQRVIGQNEAIE   49
contig 089527.IRM-030 1   ......................DEIARIISRWTGIPVARLTEGERTKLLHLEDELHKRVIGQDEGVR   45
contig 106900.IRM-030 13  IQEDLKK.KQGDNAMIKEEVTAEDIADVVSRWTGIPVSKMLQSERDKLLHLEDELHKRVIGQDEAIE  576
contig 109347.IRM-030 1   IQEDLKH.KQGDNAMIKEEVTAEDIADVVSRWTGIPVNKMLQSERDKLLNLEQELHLRVVGQEEAIA  480
contig 112201.IRM-030 1   ..............MIKEEVDAEDIADVVSRWTGIQVSKMLQSEKDKLLHLEEELHQRVIGQDEAIE   53
contig 116309.IRM-030 1   EEERIR...NEDLSLVRESVSEDEIARIVSKWTGIPVAKLTESERSKTLHLDEVLH...........   67
contig 128765.IRM-030 1   .............SLVHENVSEEEIARIISRWTGIPVAKLTESERNKTLHLDEELHKRVIGQDEGVT   54
contig 132098.IRM-030 1   EEKIAAA..KKEDSLLRDRVTDEEIARIVARWTGIPVEKLVEGEREKLLHLDDVLHKRVIGQDEAVT  223
contig 140756.IRM-030 1   EEKSVK...ESDRSLVHEAVTDDEIARIISRWTGIPVTRLTEGERAKLLTLEDQLHKRVVGQDEGVK  189
seq 15151.IRM-030 1       SEAALEAQKQQDGEGLNEQVTSAEIAEVVSAWTGVPVSKMMQGELDKLKGLEGELHKRVIGQDEAVS   68
seq 75264.IRM-030 1       ........KQGDSALIKEEVTAEDCADVVSRWTGIP...............................   76
seq 143176.IRM-030 1      ......................DDVAKVIELWTGIPAVKIRETEFVKLAGLEAALKQKVIGQDEAVH   45
seq 163948.IRM-030 1      KQAAAG..........EIQVTMDDVAKVIELWTGIPAVKIRETEYAKLASLEAELKKKIIGQDDA..   91
seq 321549.IRM-030 1      ....LKT.MQGASAMIKEEVDSEDIADVVSRWTGIPVSKMMQSEKEKLLHLESELHTRVIGQEEAIS   62
seq 436330.IRM-030 1      ...............LRDRVTDEEIARIVARWTGIPVEKLVEGEREKLLHLDDVLHQ..........   42
seq 501661.IRM-030 1      VKEKLKT.MQGASAMIKEEVDSEDIADVVSRWTGIPVSKMMQSEKEKLLHLESELHTRVI.......   79
seq 701163.IRM-030 1      .....KK.KQGDSALIKDEVTAEDIADVVSRWTGIPVSKMLQSERENLLH.................   74
seq 845795.IRM-030 1      ...SKKDENGNDNVMLKEEVDEEDIAKVVSTWTAIPVARLGTGARAKLVHLEEILHEHVIGQDEAVK   64
seq 878766.IRM-030 1      ...................VDAEDVAEVVSRWTGIPVSRMLASEREKLLHMEEELHRRVVGQDLAIA   48
seq 966940.IRM-030 1      VKKKLAD.MKHGESLIREEVTSDDIAAVVSIWTGIPVNRMMQSVRTKLLHLEDELHKRVVWQEVAIA   75
seq 1035782.IRM-030 1     IQDQLKD.TQGGDAMIKEEVTSADIADVVSRWTGIPVSKMLQS........................   74
```

logo

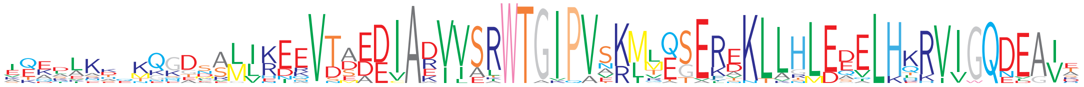

- X Asp, Glu
- X Arg, Lys, His
- X Phe, Tyr, Trp
- X Ala, Gly
- X Cys, Met
- X Ser, Thr
- X Asn, Gln
- X Leu, Val, Ile
- X Pro

```
alphaMSH.IRM-143          .............................RW.GKPV.............................    6
Ecoli-ClpB.IRM-143        ...TQL..EGKTMRLLRNKVTDAEIAEVLARWTGIPVSRMMESEREKLLRMEQELHHRVIGQNEAVD  578
contig 000421.IRM-143 1   ..QEQLKSTQGGAAMVREEVTADDIAEVVSRWTGIPVSRMMQSEREKLLHLEEELHKRVIGQDEAIT  190
contig 005972.IRM-143 1   ....................EEEIARIISRWTGIPVAKLTESERNKTLHLDEELHKRVIGQEDGVR   46
contig 014647.IRM-143 1   ..EKSV..KESDRSLVHEAVTDDEIARIISRWTGIPVTRLTEGERAKLLTLEDQLHKRVVGQDEGVK  164
contig 023387.IRM-143 1   ..EEEV..KNKDLSLVHENVSEEEIARIISRWTGIPVAKLTESERNKTLHLDEELHKR.........   73
contig 030035.IRM-143 1   ...EANNKNDKENVLLKEEVDEEDISKVVSTWTGIPVSRLSGGEREKLVHLEDILHQRVVGQDDAVK  111
contig 033429.IRM-143 1   ..EAKVK..AKDLSLVHESVTDDEIAKIVSRWTGIPVAKLNESERNKTLHLADELHKRVIGQDEGVE  129
contig 034881.IRM-143 1   ..EAKVK..AKDLSLVHESVTDDEIAKIVSRWTGIPVAKLNESERNKTLHLADELHKRVIGQDEGVE  179
contig 037029.IRM-143 1   ...EKIAAAKKEDSLLRDRVTDEEIARIVARWTGIPVEKLVEGEREKLLHLDDVLHQRVIGQDEAVT   68
contig 044192.IRM-143 1   ..EEKAEKSKTKNTLLRDKVTEEEIARIICRWTGIPVAKLM..........................  147
contig 044771.IRM-143 3   ........................ARLVARWTGIPVEKLVEGEREKLLHLDDVLHKRVIGQDEAVT   42
contig 063398.IRM-143 1   ...............VHDRVTDEEIARIISRWTGIPVAKLTEGERTKILHLDDELHKRVIGQEDGVR   52
contig 064112.IRM-143 1   QLERELPAKQ..AAAGEIQVTMDDVAKVIELWTGIPAVKIRETEYAKLASLETE.............   64
contig 066288.IRM-143 1   ...EKLAEEPQQSSLLRDKVTEEEIARIIERWTGIPVANLMEGEREKLLHLEDILHQRVVGQDEAVR   75
contig 067406.IRM-143 1   ....KIAAAKKEDSLLRDRVTDEEIARIVARWTGIPVEKLVEGEREKLLHLDDVL............   51
contig 070189.IRM-143 1   ..QDEFRAASASGAMLKEEVDAEDVAEVVSRWTGIPVSRM...........................  149
contig 074292.IRM-143 1   ..QEDLKKKQGDNAMIKEEVTAEDIADVVSRWTGIPVSKMLQSERDKLLHLEDELHKRVIGQDEAIE   68
contig 081234.IRM-143 1   ..EA....KESDDALLQEKVTVDNVSEVISRWTGIPMNKLMASEREKLLALDDTLKVRVIGQDDA..   61
contig 082643.IRM-143 1   ..EALL..KDRDLSLVRENVGDEEIALIISRWTGIPVAKLTESERNKTLHLDEELHKRVIGQDDGVT  377
contig 088472.IRM-143 1   ..EEEV..KNKDLSLVHENVSEEEIARIISRWTGIPVAKLTESERNKTLHLDEELHKRVIGQDEGVT   86
contig 096522.IRM-143 1   ..EAKAKKDGSDDSLVHENVTEEEIAKIISRWTGIPVAKLSESERQKTLNLDETLHKRVVGQDEAVT   89
contig 102803.IRM-143 1   ..EEKV..KNEDLSLVHESVTDEEIARIISRWTGIPVAKLTESERNKTLHLDEELHKRVIGQDE...  149
contig 102892.IRM-143 1   ..QEDLKHKQGDSAMIKEEVTAEDIADVVSRWTGIPVNKMLQSERDKLLHLEQELHLRVVGQDE...  128
contig 104853.IRM-143 1   ..EKKS..SNRDMSLVHESVTEEEIAKIISRWTGIPVSKLSESERQKTLNLDEQLHKRVIGQDEGVT   79
contig 118208.IRM-143 1   ELEKQTKKAQREIRRKNLSVTEDDVADVVSGWTKIPVKKLAEGEAARLKKLEATLHKRVVGQEEAVT  234
contig 119346.IRM-143 1   ...........................IISRWTGIPVAKLTETEREKTLHLDKQLHKRVIGQDEAVT   40
contig 128138.IRM-143 1   ......HKQGDNAMIKEEVTAEDIADVVSRWTGIPVNKMLQSERDKLLNLEQELHLRVVGQEEAIA   60
contig 129621.IRM-143 1   SLERKMKRNQKKQ.NEIPELTTEDAAEVVSLWTQIPVTQLTKGDMERLRHLEKELHKHVIGQDEAVN   74
contig 131246.IRM-143 1   ALEAQK...QQDGEGLNEQVTSDEIAEVVSAWTGVPVSKMMQGELDKLKGLEGELHKRVIGQDEAVS  167
```

logo

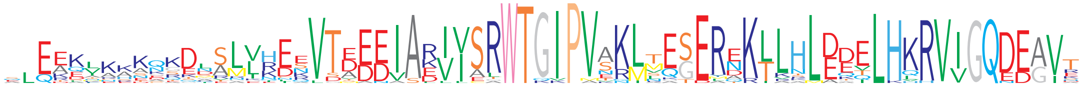

X Asp, Glu
X Arg, Lys, His
X Phe, Tyr, Trp
X Ala, Gly
X Cys, Met
X Ser, Thr
X Asn, Gln
X Leu, Val, Ile
X Pro
